# Effects of Continuous Graduated Pneumatic Compression and Intermittent Pneumatic Compression on Lower Limb Hemodynamics for VTE Prophylaxis in Arthroplasty

**DOI:** 10.1111/os.14360

**Published:** 2025-01-22

**Authors:** Binglong Li, Xuezhou Li, Weibo Zheng, Shusheng Wei, Baoqing Zhang, Jianwei Liu, Yongyuan Chen, Dan Wang, Qunshan Lu, Peilai Liu

**Affiliations:** ^1^ Department of Orthopedics Qilu Hospital of Shandong University Jinan China; ^2^ Department of Orthopedics and Sports Medicine Shengli Oilfield Central Hospital Dongying China; ^3^ Cheeloo College of Medicine Shandong University Jinan China; ^4^ Research and Development Department Shandong Zepu Medicine Technology Co. Ltd. Weifang China

**Keywords:** arthroplasty, continuous graduated pneumatic compression, hemodynamics, intermittent pneumatic compression, venous thromboembolism

## Abstract

**Objective:**

Intermittent pneumatic compression (IPC) is considered the standard of care for preventing venous thromboembolism (VTE) in the hospital setting. However, its widespread adoption after hospitalization has been limited due to its shortcomings in obstruction of venous valves and blood reflux. The objective of this study is to compare the effects of continuous graduated pneumatic compression (CGPC), a new device with a novel mechanism, and IPC on lower hemodynamics and the incidence of VTE in patients undergoing arthroplasty.

**Methods:**

We randomized 123 participants undergoing knee arthroplasty to receive either IPC or CGPC from June 2022 through August 2023. An experienced sonographer used a Doppler ultrasound scanner to obtain hemodynamic indicators of venous blood. The primary outcome was the blood velocity of the femoral vein measured by a Doppler scanner. Secondary outcomes included the hemodynamic of the femoral vein and popliteal vein, quality of life at discharge and 30 days after surgery, symptomatic and asymptomatic VTE up to 30 days, and adverse events related to the IPC and CGPC device. For statistical analyses, Student's *t*‐test, analysis of covariance, and the Mann–Whitney *U* test were used. Statistical significance was indicated with *p* < 0.05.

**Results:**

There was no significant difference in femoral vein velocity between the IPC and CGPC groups. However, CGPC demonstrated a significant increase in femoral vein flow compared to the IPC group, with a median (interquartile) increasing from 158.9 (122.9, 204.3) to 265.6 (203.3, 326.8) mL/min in the CGPC group and from 139.0 (103.3, 175.9) to 189.6 (161.4, 270.8) mL/min in the IPC group (*p* < 0.001). Similar trends were observed in popliteal vein measurements. The differences between the two groups were similar in terms of quality of life, incidence of VTE, and adverse events.

**Conclusion:**

The CGPC device provides a substantial increase in blood flow compared to the IPC device. Its safety and effectiveness have been preliminarily validated. The CGPC device presents a promising alternative for VTE prophylaxis in arthroplasty.

**Trial Registration:** Chinese Clinical Trial Registry (registration number: ChiCTR2300078201)

## Graphical abstract

We developed and manufactured an innovative mechanical device for VTE prophylaxis, named continuous graded pneumatic compression (CGPC). Its safety and effectiveness have been confirmed through validation within a patient cohort undergoing knee arthroplasty. The CGPC device demonstrates comparable venous blood velocity in the popliteal and femoral veins, but it provides a pronounced increase in blood flow compared to the intermittent pneumatic compression device, which provides an advantage in reducing venous congestion. These findings suggest that CGPC may offer a more appealing alternative approach for VTE prophylaxis in orthopedic patients.

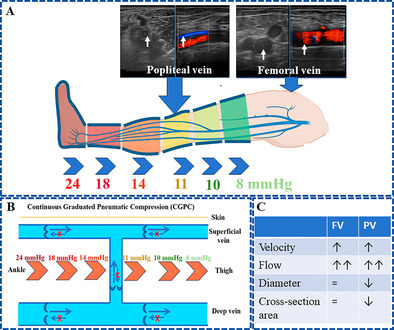

## 1 Introduction

Prophylaxis for venous thromboembolism (VTE), comprising deep vein thrombosis (DVT) and pulmonary embolism (PE), remains a crucial component of routine postoperative treatment after arthroplasty [1, 2]. In patients undergoing orthopedic surgery, the use of a tourniquet and polymethylmethacrylate bone cement, surgical manipulations, and immobilization cause pathophysiologic processes of Virchow's triad [3]. According to the Caprini risk assessment model, lower limb joint arthroplasty is considered an independent risk factor. When the Caprini score is greater than or equal to 5 points, the incidence of DVT ranges from 40% to 80%, with a mortality rate ranging from 1% to 5% [4, 5]. Thus, the American College of Chest Physicians (ACCP) classifies orthopedic patients without VTE prophylaxis at the highest risk [6].

There are two main types of VTE prophylaxis methods: mechanical and pharmacological. Mechanical methods include mobilization, intermittent pneumatic compression (IPC), venous foot pumps, and graduated compression stockings (GCS). Pharmacological approaches include low‐molecular‐weight heparin (LWGH), aspirin, vitamin K antagonists, and new oral anticoagulants [3]. Compared to pharmacological methods, mechanical VTE prophylaxis offers several advantages, including no risk of bleeding, a low incidence of clinical side effects, and no requirement for laboratory monitoring [7].

Mechanical methods have been demonstrated to decrease the risk of postoperative DVT by 50% in hospitalized patients by reducing venous stasis [8]. IPC, as a common mechanical method, applies equal pressure evenly to the lower leg. Through periodic inflation and deflation, blood and lymphatic fluid are expected to direct toward the proximal end of the limb [9, 10]. However, IPC has several limitations. On the one hand, the presence of venous valves hinders the smooth flow of blood from the lower leg, knee joint, and thigh toward the heart after compression. On the other hand, defects in venous valves, such as valvular insufficiency, can lead to venous reflux [11, 12]. Although the GCS has the advantages of greater acceptability and ease of application, it is not uncommon to encounter issues related to compliance [13].

Thus, we designed and manufactured a novel mechanical apparatus called continuous graduated pneumatic compression (CGPC), which provides an external graduated pressure gradient from the ankle to the thigh for VTE prophylaxis. This prevents the potential tourniquet effect that might occur from applying uniform pressure across the lower limb, similar to what can occur in the IPC [14].

The primary objective of this study was to compare the effects of IPC and CGPC on lower limb hemodynamics using Doppler ultrasound. Secondary objectives include profiling the adverse effects of ICP and CGPC, comparing the quality of life between ICP and CGPC, and providing a theoretical foundation for VTE prevention in orthopedic patients.

## 2 Methods

### 2.1 Ethics Approval

This study was approved by the Ethics Committee on Scientific Research of Shandong University Qilu Hospital (KYLL‐202210‐006‐1). Written informed consent was obtained from all participants.

### 2.2 Study Population

Orthopedic inpatients aged between 40 and 80 years who were undergoing arthroplasty and assessed as being at moderate or high risk of VTE (Caprini ≥ 3) were enrolled by research assistants in this study. The exclusion criteria were as follows: previous VTE; pregnancy; contraindications to IPC and CGPC, including severe peripheral neuropathy and severe peripheral arterial disease; lower limb surgery; or the use of a brace or cast.

### 2.3 Perioperative Management

**FIGURE 1:**
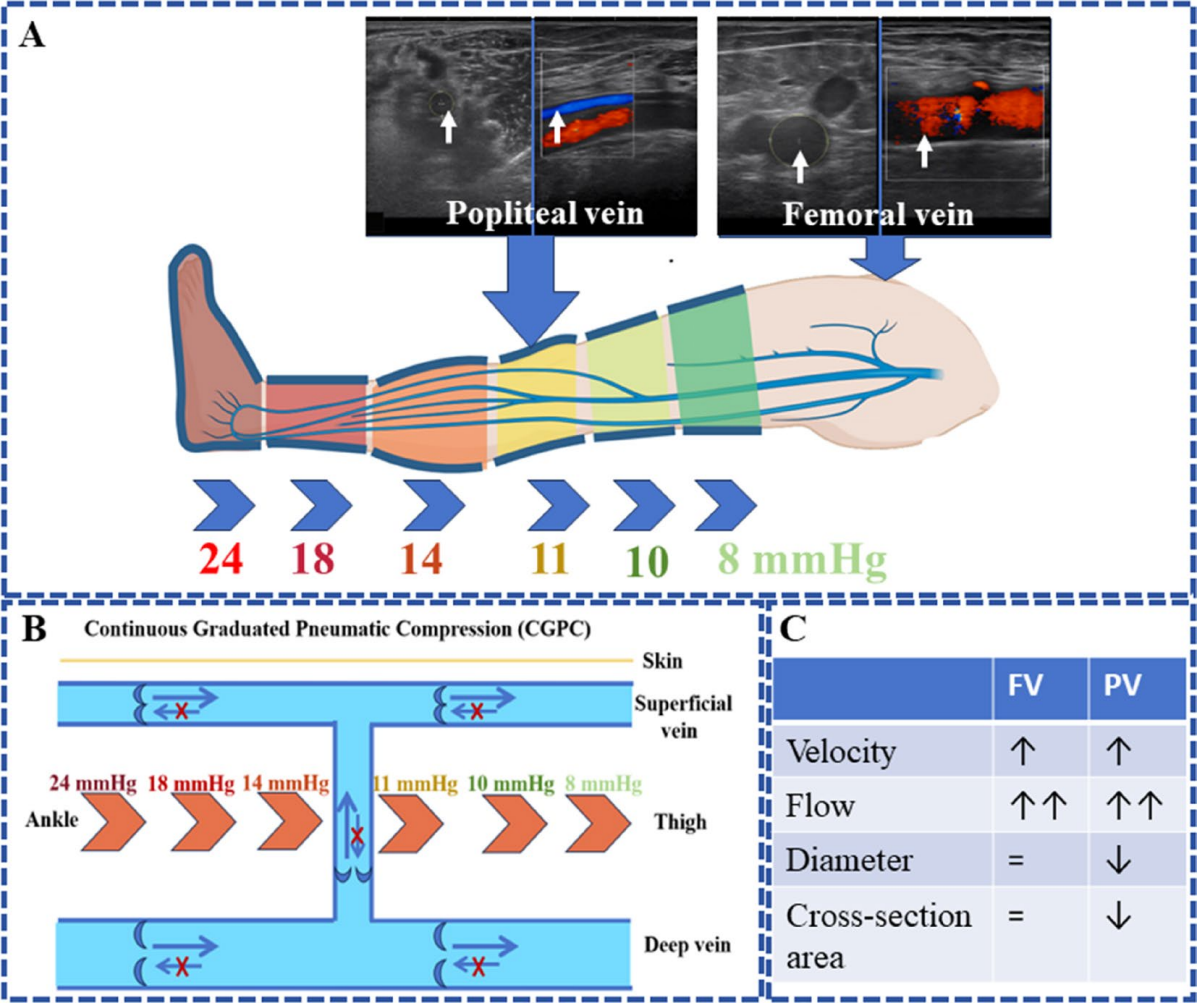
Schematic of the CGPC device. (A) Schematic of the CGPC device; (B) mechanism of action of CGPC on blood vessels; (C) hemodynamic changes in CGPC. FV, femoral vein; PV, popliteal vein; = represents no change after compression.

**FIGURE 2:**
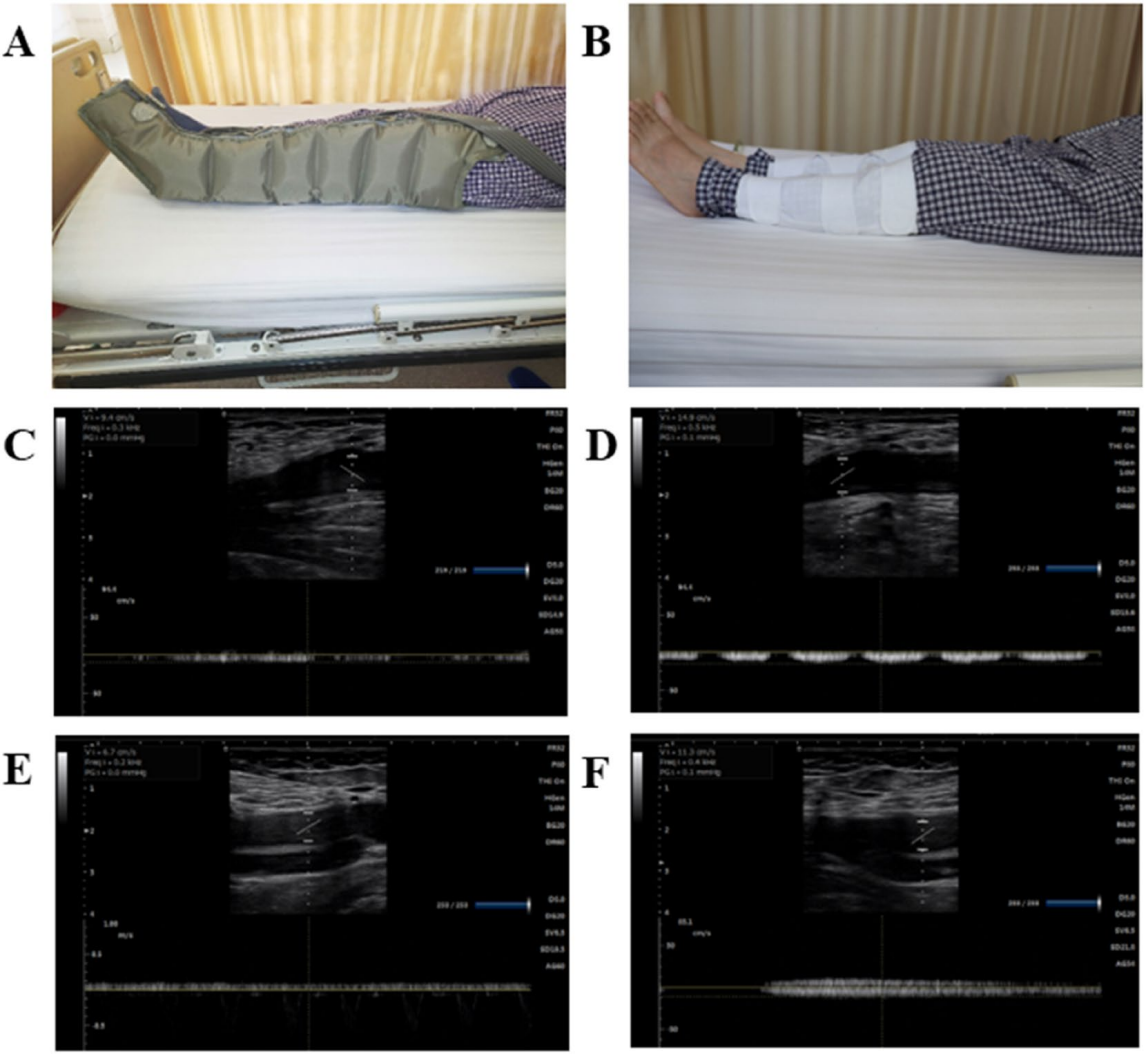
Comparison between CGPC and IPC. (A) CGPC sleeves; (B) IPC sleeves; (C) femoral vein of the CGPC at baseline; (D) femoral vein of the CGPC after compression; (E) femoral vein of the IPC at baseline; (F) femoral vein of the IPC after compression.

Participants were randomized to either receive CGPC or receive IPC (Figures 1A and 2A,B) before they underwent surgery at a ratio of 1:1 by using a computer‐generated random number. Due to the nature of the intervention, it was not feasible to blind the researcher or the patient to the study allocation. The technologist who conducted the ultrasound scans and the research assistants who collected the data were blinded to the allocation of the patients' treatments. In addition, the statistician maintained a state of blinding throughout the entire study. During hospitalization, the patient receives CGPC or IPC twice a day, with each session lasting 60 min.

After surgery, patients received a subcutaneous injection of fondaparinux sodium injection 2.5 mg once a day. Upon discharge, they were prescribed rivaroxaban tablets for 35 days postoperatively, at a dosage of 10 mg once a day. All patients wore GCS for 2 weeks following the surgery. They performed exercises, including straight leg raises and ankle pump exercises, three times a day, with each session lasting 10 min.

### 2.4 Outcomes

Participants were instructed to empty their bladder and rest in bed for half an hour before the measurement. During the entire experimental process, participants were instructed to relax, maintain calm breathing, and refrain from moving their limbs. To determine the hemodynamics of venous blood, a Doppler ultrasound scanner (SONIMAGE HS1, Shanghai Zhongke Zaiqi Medical Technology Co. Ltd., Shanghai, China) with a 4–18 MHz linear probe was used. The diameter, cross‐sectional area, velocity, and flow of the popliteal vein and femoral vein were measured at baseline, and the measurement positions were marked with a marker pen. The experimental group was subjected to CGPC for 30 min, while the control group was subjected to IPC for 30 min. Then, the above indicators were measured at the same positions. All measurements were performed by an experienced ultrasound physician.

The primary outcome of the study was the blood velocity of the femoral vein. Several studies have proposed that the extent of increase in venous blood velocity serves as a reliable hemodynamic indicator of device efficacy [15, 16, 17]. Therefore, the blood velocity of the femoral vein is considered the primary observational indication. Secondary outcomes included hemodynamics of the femoral and popliteal veins, including diameter, cross‐sectional area, velocity, and flow; symptomatic or asymptomatic VTE up to 30 days after surgery; quality of life, measured through the use of a standard health‐related quality‐of‐life instrument, namely, the EuroQol‐5 Dimensions, five‐level version (EQ‐5D‐5 L); and adverse effects of ICP and CGPC. Skin ulceration was staged according to the National Pressure Ulcer Advisory Panel classification, and the highest stage during the trial period was reported [18]. The EQ‐5D‐5 L, a widely generic and recognized tool, was employed to measure quality of life related to health. It comprises two components: a visual analog scale known as the EuroQol‐visual analog scale (EQ‐VAS) and a descriptive system. The descriptive system evaluates the participants' mobility, self‐care, regular activity, pain or discomfort, and anxiety or depression. The participants choose the option that most accurately reflects their current state of health, ranging from no problem to extreme problems. Participants are required to mark an X on the EQ‐VAS to indicate their current health status [19, 20, 21]. Any symptoms or signs of VTE, EQ‐5D‐5 L generic quality‐of‐life assessment, or adverse events were recorded by research assistants at discharge. All patients were scheduled for a follow‐up visit to the hospital for a bilateral duplex ultrasound scan of their legs at 30 days postsurgery.

### 2.5 Sample Size and Statistical Analysis

Our pilot study with Doppler ultrasound revealed a mean velocity of the femoral vein of 13 cm/s in the IPC group and a mean velocity of 14 cm/s in the experimental group. Assuming equal variance with a standard deviation of 1.4, we estimated that 43 participants per group (86 total) were required to be randomly assigned to reliably test our hypothesis, assuming a significance level (*α*) of 0.05 and a power of 90%. The specific sample size calculation was conducted using two‐sample *t*‐tests assuming equal variance. To account for follow‐up losses and incomplete data, 54 subjects were included per group (108 in total).

Statistical analysis was conducted using SPSS Statistics version 26 (International Business Machines Corporation, Arizona, USA). Parametric data are presented as the mean (SD), nonparametric data as the median (interquartile range [IQR]), and discrete variables as percentages (%). For parametric data, normality was assessed utilizing the Shapiro–Wilk test, and the data were analyzed with the covariance test or Student's *t*‐test. For nonparametric data, the Mann–Whitney *U* test was used to analyze differences. Categorical data were analyzed with the chi‐square test or Fisher's exact test. All the data were analyzed before unblinding, and *p* < 0.05 was considered to indicate statistical significance.

## 3 Results

**FIGURE 3:**
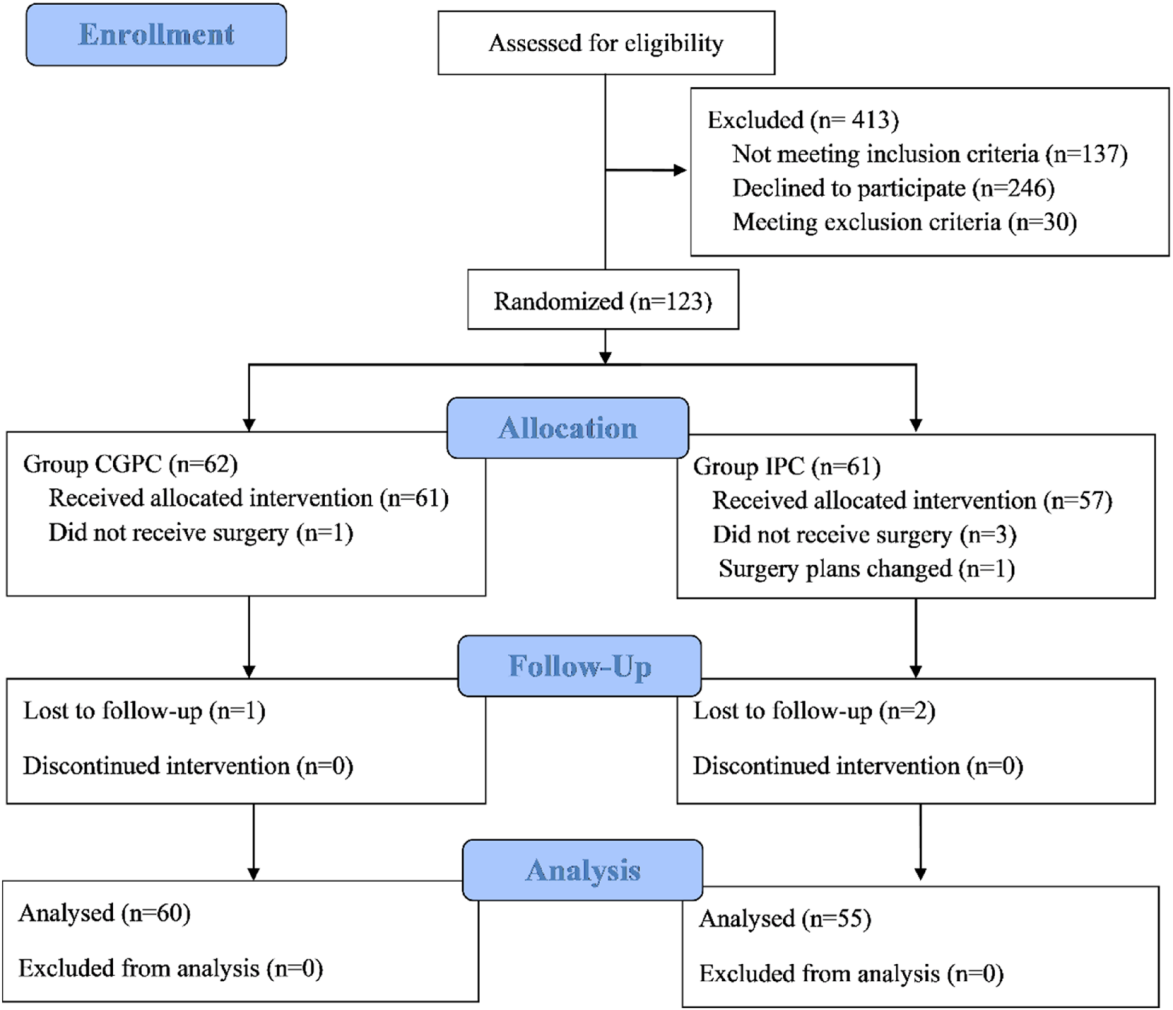
Consolidated Standard of Reporting Trials (CONSORT) flow diagram showing assessment of eligibility, enrollment, and follow‐up.

The consolidated Standard of Reporting Trials (CONSORT) flow diagram outlines the number of participants who received the designated intervention (Figure 3). From June 2022 through August 2023, a total of 536 patients were evaluated for eligibility. Of these, 413 subjects either declined participants, did not meet the inclusion criteria, or met the exclusion criteria. Thus, 123 participants were included and randomized with 62 allocated to the CGPC group and 61 to the IPC group. A total of 115 patients completed the study protocol and were included in the final analysis, with 60 in the CGPC group and 55 in the IPC group. Baseline characteristics of the patients showed no significant differences between both groups (Table 1).

**TABLE 1:** Baseline characteristics of the participants.

	IPC (*n* = 55)	CGPC (*n* = 60)	*t*	*p*
Age (years)	62.87 ± 5.62	63.80 ± 6.49	0.8166	0.416
Sex				
Male	16 (29.1%)	24 (40%)	1.505^a^	0.220
Female	39 (70.9%)	36 (60%)		
Height (m)	1.62 ± 0.74	1.62 ± 0.76	0.295	0.768
Weight (kg)	71.03 ± 10.60	72.53 ± 9.70	0.792	0.430
BMI (kg/m^2^)	27.15 ± 3.54	27.63 ± 3.27	0.758	0.450

*Note*: Values are reported as the mean (SD) or *n* (percentages).

Abbreviations: BMI, body mass index; CGPC, continuous graduated pneumatic compression; IPC, intermittent pneumatic compression.

^a^ *χ* ^2^ value.

### 3.1 Lower Limb Venous Hemodynamics

**FIGURE 4:**
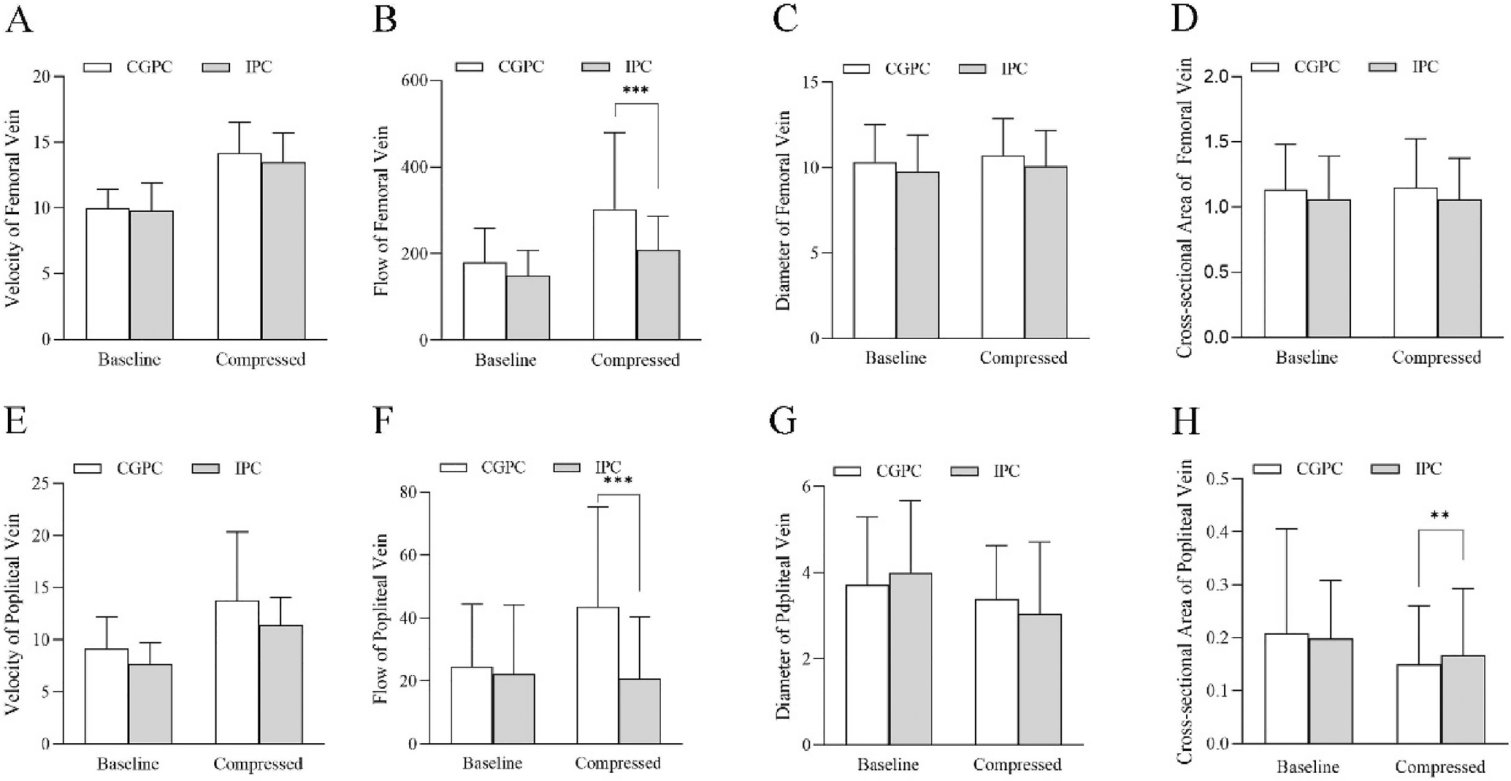
Differences in the hemodynamic indices of the femoral and popliteal veins between the CGPC and IPC groups. (A–D) represent the velocity, flow, diameter, and cross‐sectional area of the femoral vein, respectively; (E–H) represent the velocity, flow, diameter, and cross‐sectional area of the popliteal vein, respectively.

There was no significant difference in the velocity of the femoral vein between the IPC and CGPC groups (Figure 4); specifically, the median (IQR) velocity changed from 10.0 (8.3, 11.1) to 13.5 (11.9, 14.9) cm/s in the IPC group and from 9.8 (8.8, 11.3) to 14.2 (11.9, 15.8) cm/s in the CGPC group (*p* = 0.107) (Figure 2C–F). There was a statistically significant difference in the flow of the femoral vein between the CGPC group and the IPC group (Figure 4), with the median (IQR) increasing from 158.9 (122.9, 204.3) to 265.6 (203.3, 326.8) mL/min in the CGPC group and from 139.0 (103.3, 175.9) to 189.6 (161.4, 270.8) mL/min in the IPC group (*p* < 0.001). There was no significant difference in femoral vein diameter (*p* = 0.479) (Table 2). There was also no significant difference in the cross‐sectional area of the femoral vein (*p* = 0.317) (Table 2).

**TABLE 2:** Lower limb venous hemodynamic indices.

		IPC	CGPC	*F* value	*p* value^a^
Femoral vein					
Velocity (cm/s)	Baseline	10.0 (8.3, 11.1)	9.8 (8.8, 11.3)	2.637	0.107
	Compressed	13.5 (11.9, 14.9)	14.2 (11.9, 15.8)		
Flow (mL/min)	Baseline	139.0 (103.3, 175.9)	158.9 (122.9, 204.3)	13.260	0.000
	Compressed	189.6 (161.4, 270.8)	265.6 (203.3, 326.8)		
Diameter (mm)	Baseline	9.0 (8.2, 11.5)	9.8 (8.9, 10.9)	0.505	0.479
	Compressed	9.6 (8.4, 11.6)	10.3 (9.1, 11.6)		
Cross‐section area (cm^2^)	Baseline	0.98 (0.84, 1.21)	1.09 (0.85, 1.32)	1.008	0.317
	Compressed	0.99 (0.85, 1.26)	1.08 (0.88, 1.35)		
Popliteal vein					
Velocity (cm/s)	Baseline	7.5 (6.2, 9.2)	8.5 (6.5, 11.5)	0.024	0.876
	Compressed	11.1 (9.5, 13.2)	12.6 (10.2, 15.3)		
Flow (mL/min)	Baseline	15.2 (6.8, 31.3)	29.6 (5.6, 41.8)	14.549	0.000
	Compressed	14.5 (5.5, 29.6)	46.7 (10.9, 72.8)		
Diameter (mm)	Baseline	3.8 (2.5, 5.5)	3.3 (2.7, 4.6)	0.160	0.690
	Compressed	2.7 (1.7, 4.3)	3.5 (2.2, 4.4)		
Cross‐section area (cm^2^)	Baseline	0.19 (0.11, 0.30)	0.20 (0.06, 0.26)	12.878	0.001
	Compressed	0.12 (0.06, 0.22)	0.15 (0.05, 0.22)		

*Note*: Values are reported as the median (IQR).

Abbreviations: CGPC, continuous graduated pneumatic compression; IPC, intermittent pneumatic compression.

^a^ Variables were compared between the two trial groups with analysis of covariance.

The median (IQR) flow of the popliteal vein was significantly different between the two groups (Figure 4). In the CGPC group, the median (IQR) flow changed from 29.6 (5.6, 41.8) to 46.7 (10.9, 72.8) mL/min, while in the IPC group, it changed from 15.2 (6.8, 31.3) to 14.5 (5.5, 29.6) mL/min (*p* < 0.001). The IPC group exhibited a lower median cross‐sectional area (with IQR) than did the CGPC group (*p* = 0.001). There was no significant difference in the velocity of the popliteal vein (Table 2). Similarly, there was no statistically significant difference in the diameter of the popliteal vein between the two groups.

### 3.2 Quality of Life

**FIGURE 5:**
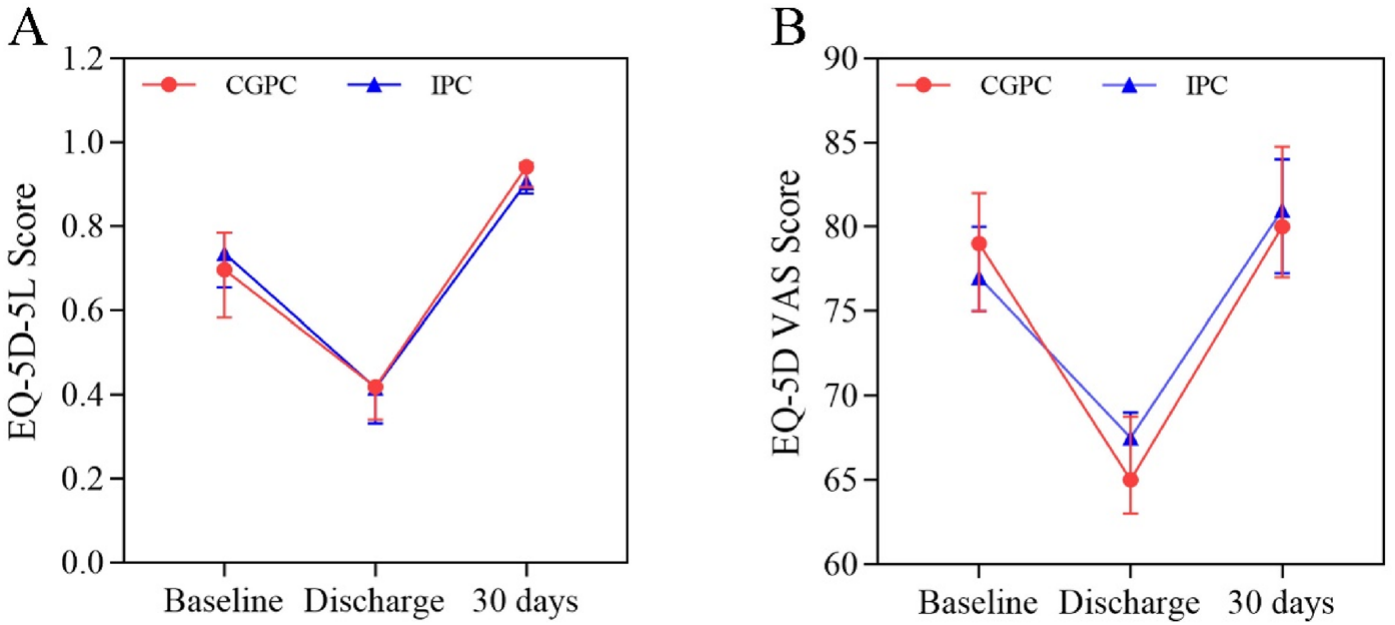
Quality of life at baseline, discharge, and 30 days postoperation. (A) EQ‐5D‐5 L scores at baseline, discharge, and 30 days postoperation; (B) EQ‐5D VAS scores at baseline, discharge, and 30 days postoperation.

Concerning quality of life, the EQ‐5D‐5 L scores were comparable between the two groups at baseline. There was no evidence of a difference in the EQ‐5D‐5 L scores across the follow‐up time points for the overall population (Figure 5A). There was no difference in the EQ‐5D VAS score at baseline, discharge, or 30 days postoperation (Figure 5B).

### 3.3 Incidence of Venous Thromboembolism

DVT occurred in 1 out of 56 patients (1.8%) in the CGPC group and in 1 out of 48 participants (2.1%) in the IPC group (relative risk, 0.86; 95% confidence interval [CI], 0.06 to 13.34). There was no discernible difference in the treatment effect concerning incident DVT. Neither group of patients experienced PE within 30 days postoperation (Table 3).

**TABLE 3:** Quality‐of‐life outcomes and details of venous thromboembolism patients.

		IPC	CGPC	*t* value	*p* value
Quality of life					
EQ‐5D‐5 L	Baseline	0.74 (0.65, 0.78)	0.69 (0.57, 0.78)	−1.054	0.294
	Discharge	0.42 (0.34, 0.42)	0.42 (0.34, 0.43)	1.610	0.110
	30 days	0.90 (0.88, 0.94)	0.94 (0.89, 0.95)	1.009	0.315
EQ‐5D VAS	Baseline	77.0 (75.0, 80.0)	79.0 (75.0, 82.8)	1.278	0.204
	Discharge	66.0 (65.0, 69.0)	65.0 (63.0, 68.0)	−1.781	0.078
	30 days	81.0 (77.3, 84.0)	80.0 (77.0, 84.8)	0.229	0.819
Venous thromboembolism^a^					
Incident distal lower‐limb DVT		1 (2.1%)	1 (1.8%)	0.000	1.000
Relative risk (95% CI)		0.86 (0.06, 13.34)		NA	NA
Pulmonary embolism		0	0	NA	NA

Abbreviations: CGPC, continuous graduated pneumatic compression; DVT, deep vein thrombosis; EQ‐5D‐5 L, EuroQol five‐dimensional five‐point scale; EQ‐5D VAS, EuroQol five‐dimensional visual analog scale; IPC, intermittent pneumatic compression.

^a^ The incidence of venous thromboembolism within 30 days.

### 3.4 Safety and Adverse Events

Safety was assessed in the intention‐to‐treat population (Table 4). Reports of leg discomfort were noted in 3 out of 55 patients (5.5%) who received IPC and 2 out of 60 patients (3.3%) who received CGPC. Skin blistering occurred in two patients (3.6%) who underwent IPC treatment. Pruritus occurred in 1.8% of the patients receiving IPC and in 3.3% of the patients receiving CGPC. However, they were unrelated to either IPC or CGPC, indicating a correlation with adhesive allergy. Additionally, the percentages of patients experiencing lower‐limb skin injury or ischemia did not show a notable difference between the IPC and CGPC groups. Lower‐limb skin injuries were not directly caused by continuous graduated pneumatic compression devices or IPC devices. Instead, they were attributed to skin sensitivity and the tearing of the dressing caused by the adhesive. There were no reports of serious adverse events or PE. Furthermore, in both groups, there was no occurrence of poor wound healing.

**TABLE 4:** Complications and adverse events as treated.

	IPC (*N* = 55)	CGPC (*N* = 60)	*χ* ^2^ value	*p* value
Discomfort in legs	3	2	0.010	0.921
Blistering of skin	1	0	0.002	0.965
Rash	0	0	NA	NA
Pruritus	1	2	0.000	1.000
Lower‐limb skin injury^a^				
Stage I: nonblanchable erythema	1	2	0.000	1.000
Stage II: partial‐thickness ulceration	0	0	NA	NA
Stage III or IV: full‐thickness skin or tissue loss	0	0	NA	NA
Serious adverse events^b^	0	0	NA	NA
Poor wound healing	0	0	NA	NA

Abbreviations: CGPC, continuous graduated pneumatic compression; IPC, intermittent pneumatic compression; NA, not applicable.

^a^ Skin ulceration was staged according to the National Pressure Ulcer Advisory Panel classification, and the highest stage during the trial period was reported.

^b^ Serious adverse events were defined as skin pressure ulcers of stage III or IV or ischemia due to IPC or CGPC.

## 4 Discussion

In the present study, although both the IPC and CGPC groups exhibited a significant increase in blood flow in the femoral and popliteal veins, the CGCP group exhibited a more notable increase in blood flow than did the IPC group. However, there was no statistically significant difference in the increase in blood velocity between the two groups.

### 4.1 Analysis of Main Findings

Although the exact mechanisms of postoperative DVT are not fully understood, it is widely acknowledged that a decrease in venous velocity and an accumulation of blood in the calf veins are significant contributing factors. External compression effectively reduces the cross‐sectional area of the venous system, thereby augmenting the linear velocity [22]. Our study revealed a notable surge in blood flow velocity and volume with both the popliteal vein and femoral vein compared to the baseline. Additionally, there was a reduction in the cross‐sectional area of the popliteal region, which was consistent with prior research [2, 23, 24, 25, 26]. Thus, there were sound theoretical grounds for believing that this was an effective method of prophylaxis.

While there was no notable difference in the increase in blood velocity in either the femoral vein or popliteal vein between the two groups, there was a significant increase in the mean velocity compared to that at baseline, which is consistent with previous studies. Espeit and Lapole [27] reported that using GCS induces significantly greater venous blood velocity in the popliteal vein than does the control intervention. Several studies [2, 28, 29] have indicated that pneumatic compression leads to a significant increase in femoral peak flow velocity compared to baseline, and it reduces major bleeding events. Sakai et al. [30] found that the use of an IPC device significantly increased the peak velocity, which indicated that IPC may be valuable in the prevention of DVT during the perioperative period. The study conducted by Zuj et al. [25] demonstrated that applying intermittent compression during the diastolic phase of the cardiac cycle enhanced the action of the muscle pump, resulting in increased superficial femoral artery blood flow.

### 4.2 The Appropriate Gradient Pressures of the CGPC

Currently, there is no consensus on how pressure should be distributed along the leg to effectively support venous pumps. While some literature has emphasized the importance of gradient compression, there has been a lack of data regarding this concept in stimulating the calf muscle pump [2, 13, 22]. The gradient pressures of the CGPC device on the ankle, lower calf, upper calf, knee, lower thigh, and upper thigh were 24, 18, 14, 11, 10, and 8 mmHg, respectively, consistent with previous studies [14, 31]. Lawrence and colleagues [14] utilized technetium‐99 to measure venous blood velocity by tracking the time it took for a bolus to travel from the injection site to the femoral vein. Their findings revealed that a pressure range of 18–8 mmHg (from the ankle to the thigh) led to a significant increase in the mean deep venous velocity without causing any subsequent impairment of either calf muscle blood flow or subcutaneous tissue perfusion. However, when the pressure range was greater than 30–12 mmHg (from the ankle to the thigh), the mean velocity increased, but a progressive decrease in calf subcutaneous tissue flow occurred. GCS, utilized for DVT prevention, exert a compression force ranging from 18 to 18 mmHg at the ankle, gradually decreasing to 2–8 mmHg at the mid‐thigh. This pressure gradient has demonstrated effectiveness in preventing DVT [31, 32]. Yamaguchi et al. [33] found that a pressure of 30 mmHg at the ankle led to an increase in popliteal vein flow, while a pressure range of 40–70 mmHg was found to decrease blood flow.

### 4.3 Potential Mechanisms of CGPC

The following are potential mechanisms for a greater increase in blood flow in the CGPC than in the IPC. The pressure gradient guarantees that blood flows toward the heart, decreases elevated hydrostatic pressure and restores a balanced pressure between the deep and superficial veins, preventing any reverse flow downward toward the foot or sideways into the superficial veins [13, 34, 35] (Figure 1B). Graduated compression can lead to a reduction in the diameter and cross‐sectional area of blood vessels, thereby increasing both the speed and volume of blood flow [13] (Figure 1C). It can enhance the skeletal muscle pump, reverse venous hypertension, facilitate lymphatic drainage, and promote venous return [36]. Thus, compared with previous IPC devices, the CGPC device, which incorporates innovative mechanical features, enables greater movement of venous blood through the lower extremity by increasing the mean venous blood velocity and flow.

### 4.4 Limitations and Prospect

We acknowledge that our trial has several limitations. First, a larger sample size is necessary to accurately reflect the potential differences in the occurrence rates of VTE between the two groups. Second, this study did not assess whether both treatments had a systemic effect on blood coagulability or fibrinolytic activity. Further studies are required to investigate whether IPC and CGPC have a systemic effect on hematological parameters. In the future, the research will focus on the effects of CGPC with different pressure gradients on lower extremity hemodynamics and its clinical effectiveness in preventing deep vein thrombosis.

## 5 Conclusion

In conclusion, we developed and manufactured an innovative mechanical device for VTE prophylaxis, named continuous graduated pneumatic compression (CGPC). Its safety and effectiveness have been confirmed through validation within a patient cohort undergoing knee arthroplasty. The CGPC device demonstrates comparable venous blood velocity in the popliteal and femoral veins, but it provides a pronounced increase in blood flow compared to the IPC device, which provides an advantage in reducing venous congestion. These findings suggest that CGPC may offer a more appealing alternative approach for VTE prophylaxis in orthopedic patients.

## Author Contributions

Binglong Li mainly completed the study design and conduct, data collection and analysis, and a draft of the manuscript. Xuezhou Li completed a study design and conducted, data collection and analysis, and revision of the manuscript. Weibo Zheng and Shusheng Wei completed the execution of the trial, data collection, and analysis. Baoqing Zhang completed the execution of the trial, Jianwei Liu, Yongyuan Chen, and Dan Wang completed the design of CGPC and analysis, Qunshan Lu and Peilai Liu completed the study concept, study design, and revision of the manuscript. All authors read and approved the final manuscript.

## Conflicts of Interest

The authors declare no conflicts of interest.

## References

[1] A. T. Cohen , V. F. Tapson , J. F. Bergmann , et al., “Venous Thromboembolism Risk and Prophylaxis in the Acute Hospital Care Setting (ENDORSE Study): A Multinational Cross‐Sectional Study,” Lancet 371, no. 9610 (2008): 387–394. 18242412 10.1016/S0140-6736(08)60202-0

[2] D. F. Amanatullah , H. N. Shah , B. Johnson , and J. Wall , “Mechanical Compression Augments Venous Flow Equal to Intermittent Pneumatic Compression,” Journal of Orthopaedic Research 38, no. 11 (2020): 2390–2395. 32175638 10.1002/jor.24664

[3] D. A. Flevas , P. D. Megaloikonomos , L. Dimopoulos , E. Mitsiokapa , P. Koulouvaris , and A. F. Mavrogenis , “Thromboembolism Prophylaxis in Orthopaedics: An Update,” EFORT Open Reviews 3, no. 4 (2018): 136–148. 29780621 10.1302/2058-5241.3.170018 PMC5941651

[4] V. Bahl , H. M. Hu , P. K. Henke , T. W. Wakefield , D. A. Campbell, Jr. , and J. A. Caprini , “A Validation Study of a Retrospective Venous Thromboembolism Risk Scoring Method,” Annals of Surgery 251, no. 2 (2010): 344–350. 19779324 10.1097/SLA.0b013e3181b7fca6

[5] J. A. Caprini , J. I. Arcelus , J. H. Hasty , A. C. Tamhane , and F. Fabrega , “Clinical Assessment of Venous Thromboembolic Risk in Surgical Patients,” Seminars in Thrombosis and Hemostasis 17, no. Suppl 3 (1991): 304–312. 1754886

[6] Y. Falck‐Ytter , C. W. Francis , N. A. Johanson , et al., “Prevention of VTE in Orthopedic Surgery Patients: Antithrombotic Therapy and Prevention of Thrombosis, 9th Ed: American College of Chest Physicians Evidence‐Based Clinical Practice Guidelines,” Chest 141, no. 2 Suppl (2012): e278S–e325S. 22315265 10.1378/chest.11-2404 PMC3278063

[7] W. H. Geerts , G. F. Pineo , J. A. Heit , et al., “Prevention of Venous Thromboembolism: The Seventh ACCP Conference on Antithrombotic and Thrombolytic Therapy,” Chest 126, no. 3 Suppl (2004): 338s–400s. 15383478 10.1378/chest.126.3_suppl.338S

[8] J. Urbankova , R. Quiroz , N. Kucher , and S. Z. Goldhaber , “Intermittent Pneumatic Compression and Deep Vein Thrombosis Prevention. A Meta‐Analysis in Postoperative Patients,” Thrombosis and Haemostasis 94, no. 6 (2005): 1181–1185. 16411391 10.1160/TH05-04-0222

[9] B. Wang , Y. Wang , Z. Sun , et al., “Multiple Blood Flow Surges During Intermittent Pneumatic Compression: The Origins and Their Implications,” Journal of Biomechanics 143 (2022): 111264. 36055052 10.1016/j.jbiomech.2022.111264

[10] W. Lee , J. H. Seo , H. B. Kim , et al., “Investigation of Blood Flow During Intermittent Pneumatic Compression and Proposal of a New Compression Protocol,” Clinical and Applied Thrombosis/Hemostasis 24, no. 2 (2018): 338–347. 28301905 10.1177/1076029616683044 PMC6714691

[11] K. Kappa‐Markovi , H. Jalaie , H. Özhan‐Hasan , M. Deges , and K. Rass , “Intermittent Pneumatic Compression After Varicose Vein Surgery,” Journal of Vascular Surgery. Venous and Lymphatic Disorders 9, no. 6 (2021): 1526–1534.e2. 33667741 10.1016/j.jvsv.2021.02.011

[12] A. J. Comerota , “Intermittent Pneumatic Compression: Physiologic and Clinical Basis to Improve Management of Venous Leg Ulcers,” Journal of Vascular Surgery 53, no. 4 (2011): 1121–1129. 21050701 10.1016/j.jvs.2010.08.059

[13] C. S. Lim and A. H. Davies , “Graduated Compression Stockings,” Canadian Medical Association Journal 186, no. 10 (2014): E391–E398. 24591279 10.1503/cmaj.131281 PMC4081237

[14] D. Lawrence and V. V. Kakkar , “Graduated, Static, External Compression of the Lower Limb: A Physiological Assessment,” British Journal of Surgery 67, no. 2 (1980): 119–121. 7362940 10.1002/bjs.1800670214

[15] J. L. Berliner , P. A. Ortiz , Y. Y. Lee , T. T. Miller , and G. H. Westrich , “Venous Hemodynamics After Total Hip Arthroplasty: A Comparison Between Portable vs Stationary Pneumatic Compression Devices and the Effect of Body Position,” Journal of Arthroplasty 33, no. 1 (2018): 162–166. 28927565 10.1016/j.arth.2017.08.005

[16] B. J. Broderick , S. O'Connell , S. Moloney , et al., “Comparative Lower Limb Hemodynamics Using Neuromuscular Electrical Stimulation (NMES) Versus Intermittent Pneumatic Compression (IPC),” Physiological Measurement 35, no. 9 (2014): 1849–1859. 25154429 10.1088/0967-3334/35/9/1849

[17] H. Jawad , D. S. Bain , H. Dawson , K. Crawford , A. Johnston , and A. Tucker , “The Effectiveness of a Novel Neuromuscular Electrostimulation Method Versus Intermittent Pneumatic Compression in Enhancing Lower Limb Blood Flow,” Journal of Vascular Surgery: Venous and Lymphatic Disorders 2, no. 2 (2014): 160–165. 26993181 10.1016/j.jvsv.2013.10.052

[18] J. Black , M. M. Baharestani , J. Cuddigan , et al., “National Pressure Ulcer Advisory Panel's Updated Pressure Ulcer Staging System,” Advances in Skin & Wound Care 20, no. 5 (2007): 269–274. 17473563 10.1097/01.ASW.0000269314.23015.e9

[19] N. Luo , G. Liu , M. Li , H. Guan , X. Jin , and K. Rand‐Hendriksen , “Estimating an EQ‐5D‐5L Value Set for China,” Value in Health 20, no. 4 (2017): 662–669. 28408009 10.1016/j.jval.2016.11.016

[20] B. A. van Hout and J. W. Shaw , “Mapping EQ‐5D‐3L to EQ‐5D‐5L,” Value in Health 24, no. 9 (2021): 1285–1293. 34452708 10.1016/j.jval.2021.03.009

[21] Y. S. Feng , T. Kohlmann , M. F. Janssen , and I. Buchholz , “Psychometric Properties of the EQ‐5D‐5L: A Systematic Review of the Literature,” Quality of Life Research 30, no. 3 (2021): 647–673. 33284428 10.1007/s11136-020-02688-y PMC7952346

[22] N. Labropoulos , K. K. Giuliano , A. J. Tafur , and J. A. Caprini , “Comparison of a Nonpneumatic Device to Four Currently Available Intermittent Pneumatic Compression Devices on Common Femoral Blood Flow Dynamics,” Journal of Vascular Surgery: Venous and Lymphatic Disorders 9, no. 5 (2021): 1241–1247. 33540132 10.1016/j.jvsv.2021.01.008

[23] Z. Zhuang , Y. Wang , Y. Yao , Y. Shen , D. Chen , and Q. Jiang , “The Impact of Graduated Compression Stockings on Calf‐Vein Deformation and Blood Velocity in Patients Awaiting Total Knee Arthroplasty,” BMC Musculoskeletal Disorders 22, no. 1 (2021): 722. 34425810 10.1186/s12891-021-04603-z PMC8381553

[24] K. A. Zuj , C. N. Prince , R. L. Hughson , and S. D. Peterson , “Enhanced Muscle Blood Flow With Intermittent Pneumatic Compression of the Lower Leg During Plantar Flexion Exercise and Recovery,” Journal of Applied Physiology 124, no. 2 (2018): 302–311. 29122964 10.1152/japplphysiol.00784.2017 PMC5867371

[25] K. A. Zuj , C. N. Prince , R. L. Hughson , and S. D. Peterson , “Superficial Femoral Artery Blood Flow With Intermittent Pneumatic Compression of the Lower Leg Applied During Walking Exercise and Recovery,” Journal of Applied Physiology 127, no. 2 (2019): 559–567. 31268826 10.1152/japplphysiol.00656.2018

[26] K. Nakanishi , N. Takahira , M. Sakamoto , M. Yamaoka‐Tojo , M. Katagiri , and J. Kitagawa , “Effects of Intermittent Pneumatic Compression of the Thigh on Blood Flow Velocity in the Femoral and Popliteal Veins: Developing a New Physical Prophylaxis for Deep Vein Thrombosis in Patients With Plaster‐Cast Immobilization of the Leg,” Journal of Thrombosis and Thrombolysis 42, no. 4 (2016): 579–584. 27486017 10.1007/s11239-016-1403-y

[27] L. Espeit and T. Lapole , “Effects of Graduated Compression Stockings, Local Vibration and Their Combination on Popliteal Venous Blood Velocity,” Phlebology 35, no. 7 (2020): 505–512. 31973631 10.1177/0268355520902000

[28] J. Wall , E. Johnson , B. Johnson , A. Singh , R. Shaheen , and T. Fogarty , “A Pilot Study of Venous Flow Augmentation Using a Novel Mechanical Graded Intermittent Sequential Compression Device for Venous Insufficiency,” Journal of Vascular Surgery: Venous and Lymphatic Disorders 7, no. 2 (2019): 217–221. 30612969 10.1016/j.jvsv.2018.10.018

[29] D. Wang , F. Bao , Q. Li , Y. Teng , and J. Li , “Semiautomatic Intermittent Pneumatic Compression Device Applied to Deep Vein Thrombosis in Major Orthopedic Surgery,” Biomedical Engineering Online 17, no. 1 (2018): 78. 29903003 10.1186/s12938-018-0513-5 PMC6002995

[30] K. Sakai , N. Takahira , K. Tsuda , and A. Akamine , “Effects of Intermittent Pneumatic Compression on Femoral Vein Peak Venous Velocity During Active Ankle Exercise,” Journal of Orthopaedic Surgery (Hong Kong) 29, no. 1 (2021): 2309499021998105. 33641535 10.1177/2309499021998105

[31] R. Liu , T. T. Lao , Y. L. Kwok , Y. Li , and M. T. Ying , “Effects of Graduated Compression Stockings With Different Pressure Profiles on Lower‐Limb Venous Structures and Haemodynamics,” Advances in Therapy 25, no. 5 (2008): 465–478. 18523736 10.1007/s12325-008-0058-2

[32] J. L. Davies , “Graduated Compression Stockings in the Prevention of Postoperative Deep Vein Thrombosis,” British Journal of Surgery 77, no. 12 (1990): 1435–1436. 10.1002/bjs.1800771236 2276035

[33] K. Yamaguchi , H. Gans , Y. Yamaguchi , and S. Hagisawa , “External Compression With Elastic Bandages: Its Effect on the Peripheral Blood Circulation During Skin Traction,” Archives of Physical Medicine and Rehabilitation 67, no. 5 (1986): 326–331. 3707318

[34] J. Shalhoub , J. Norrie , C. Baker , et al., “Graduated Compression Stockings as an Adjunct to Low Dose Low Molecular Weight Heparin in Venous Thromboembolism Prevention in Surgery: A Multicentre Randomised Controlled Trial,” European Journal of Vascular and Endovascular Surgery 53, no. 6 (2017): 880–885. 28396238 10.1016/j.ejvs.2017.02.013

[35] S. J. Palfreyman and J. A. Michaels , “A Systematic Review of Compression Hosiery for Uncomplicated Varicose Veins,” Phlebology 24, no. Suppl 1 (2009): 13–33. 19307438 10.1258/phleb.2009.09s003

[36] C. Moffatt , “Variability of Pressure Provided by Sustained Compression,” International Wound Journal 5, no. 2 (2008): 259–265. 18494631 10.1111/j.1742-481X.2008.00470.x PMC7951751

